# Metagenomic shifts in mucus, tissue and skeleton of the coral *Balanophyllia europaea* living along a natural CO_2_ gradient

**DOI:** 10.1038/s43705-022-00152-1

**Published:** 2022-08-05

**Authors:** Giorgia Palladino, Erik Caroselli, Teresa Tavella, Federica D’Amico, Fiorella Prada, Arianna Mancuso, Silvia Franzellitti, Simone Rampelli, Marco Candela, Stefano Goffredo, Elena Biagi

**Affiliations:** 1 Unit of Microbiome Science and Biotechnology, Department of Pharmacy and Biotechnology, University of Bologna, via Belmeloro 6, 40126 Bologna, Italy; 2 Fano Marine Center, The Inter-Institute Center for Research on Marine Biodiversity, Resources and Biotechnologies, viale Adriatico 1/N, 61032 Fano, Pesaro Urbino Italy; 3 Marine Science Group, Department of Biological, Geological and Environmental Sciences, University of Bologna, via Selmi 3, 40126 Bologna, Italy; 4 Animal and Environmental Physiology Laboratory, Department of Biological, Geological and Environmental Sciences, University of Bologna, via Sant’Alberto 163, 48123 Ravenna, Italy

**Keywords:** Metagenomics, Microbial ecology, Climate-change ecology

## Abstract

Using the Mediterranean coral *Balanophyllia europaea* naturally growing along a pH gradient close to Panarea island (Italy) as a model, we explored the role of host-associated microbiomes in coral acclimatization to ocean acidification (OA). Coral samples were collected at three sites along the gradient, mimicking seawater conditions projected for 2100 under different IPCC (The Intergovernmental Panel on Climate Change) scenarios, and mucus, soft tissue and skeleton associated microbiomes were characterized by shotgun metagenomics. According to our findings, OA induced functional changes in the microbiomes genetic potential that could mitigate the sub-optimal environmental conditions at three levels: i. selection of bacteria genetically equipped with functions related to stress resistance; ii. shifts in microbial carbohydrate metabolism from energy production to maintenance of cell membranes and walls integrity; iii. gain of functions able to respond to variations in nitrogen needs at the holobiont level, such as genes devoted to organic nitrogen mobilization. We hence provided hypotheses about the functional role of the coral associated microbiome in favoring host acclimatation to OA, remarking on the importance of considering the crosstalk among all the components of the holobiont to unveil how and to what extent corals will maintain their functionality under forthcoming ocean conditions.

## Introduction

Scleractinian corals live in close association with a diverse array of phylogenetically disparate microorganisms, including endocellular photoautotrophic dinoflagellate symbionts (belonging to the Symbiodiniaceae family) and complex communities of bacteria, archaea, viruses, and unicellular eukaryotes (*i.e*., microbiomes). Indeed, each coral anatomic compartment (e.g. surface mucus, soft tissue, and skeleton) constitutes a microhabitat characterized by specific conditions supporting different micro-ecosystems (reviewed in ref. [1]). The consortium of coexisting pluri- and unicellular organisms is termed “coral holobiont”, whose microbial counterpart is believed to maintain organismal function under varying environmental conditions [2, 3]. Indeed, complex bacterial communities inhabiting coral mucus, tissue, and skeleton exert a crucial role in ensuring the health and survival of the coral as they provide their host with a variety of functions, such as assistance in recovering and recycling of nutrients (carbon, nitrogen and sulfur, but also vitamins and essential amino acids), protection against pathogens invasion, and production of chemicals that drive larval settlement (as suggested by ref. [4–9]). Besides being tightly related to host phylogeny, the composition and metabolism of coral microbiomes change temporally (across seasons and along coral lifespan), spatially (across the compartments defined by the coral anatomy), and in response to environmental variations [1, 10, 11]. Shifts in coral microbiome composition in response to environmental changes may affect the co-metabolic networks, possibly contributing to acclimatization of the coral holobiont. Indeed, microbial communities as a whole have the possibility to acclimatize faster to environmental changes than their metazoan host, thanks to their greater genetic diversity, shorter generational time, and remarkable metabolic potential [12, 13]. Such propensity for a fast response to environmental changes has been investigated as possibly involved in the acclimatization and adaptation of the coral holobiont to climate change-related phenomena, such as ocean warming and ocean acidification (OA) [2, 9, 12, 14, 15].

Ocean acidity has increased worldwide by 25–30% (0.1 pH units) since the beginning of the nineteenth century, and it is expected to drop by a further 0.29 pH units by 2080–2100 [15, 16]. OA is expected to alter the survival, growth, and reproduction of key components of marine ecosystems, especially calcifying species [17], both at microbial and multicellular levels [18–20]. Thus, it is of outmost importance, for projecting ecological processes in the forthcoming oceans, to understand how OA will affect the microbiome of important ecosystem forming organisms such as corals [21]. In this context, natural underwater CO_2_ seeps represent a precious study system to understand how corals will respond to OA [22, 23]. The Mediterranean Sea, which will likely be one of the most affected seas by climate change [24], hosts naturally acidified shallow sites with relatively stable underwater CO_2_ emission at ambient temperature with no detection of toxic compounds, that have been recognized as fundamental study models for OA [25–27].

To date, few studies have explored coral microbiome variations in natural coral populations at CO_2_ seeps [6, 28–31], showing different microbiome responses depending on the host species. For instance, at natural CO_2_ seeps in Papua New Guinea, the endolithic community associated with massive *Porites* spp. does not change with pH [6, 32], while large shifts in tissue-associated bacterial communities were found in *Acropora millepora* and *Porites cylindrica* [29]. Indeed, as recently highlighted by Shore and colleagues [31], the response to decreasing pH of the microbiome associated to *Porites* corals seems to be species-specific and does not reflect a breakdown in bacteria-host symbiosis. In the Mediterranean coral *Astroides calycularis* growing at the Ischia CO_2_ vents, the mucus-associated microbiome was more affected by acidification than soft tissue and skeleton, with a general increase in subdominant bacterial groups with OA, some of which may be involved in the nitrogen cycle [30].

The target species of the present study is the solitary Mediterranean coral *Balanophyllia europaea* that naturally lives along a pH gradient generated by an underwater volcanic crater located close to Panarea Island (Italy). *B. europaea* is a temperate, zooxanthellate, scleractinian coral, widespread in the Mediterranean Sea, where it thrives on rocky substrates at a depth of 0–50 m [33].

To date, the majority of the studies aimed at understanding the involvement of coral microbiomes in acclimatization to future acidified water conditions were focused on tropical and subtropical corals [6, 34–36]. However, temperate species, such as *B. europaea*, might represent a model for more pronounced acclimatization capability, being exposed to a twice as high range of seasonal temperature fluctuations and intrinsically more capable to accommodate environmental variations (as suggested by [37]). Here, we focus on the bacterial component of the *B. europaea* holobiont and on the variations in the metabolic potential of the microbial communities residing in surface mucus, soft tissue, and skeleton to the decreasing pH. The CO_2_ seep near Panarea Island (Italy) from which samples were taken is an underwater crater at 10 m depth releases persistent gaseous emissions (98–99% CO_2_ without instrumentally detectable toxic compounds), resulting in a stable pH gradient at ambient temperature that has been characterized in detail [25, 26, 38]. Sampling sites along this gradient match mean pH values projected for 2100 under different IPCC scenarios [39].

Since a considerable amount of research showed an overall acclimatization of this model organisms to low pH conditions, principally through the homeostatic balance of several physiological parameters e.g. gross calcification rate, calcifying fluid pH, skeletal calcium carbonate polymorph, aragonite fiber thickness, skeletal hardness and organic matrix content [25, 26, 40], here we aim at exploring the possible role of the coral-associated microbiomes in this acclimatization process. Specifically, to verify our hypothesis, we combined 16S rRNA gene sequencing and shotgun metagenomics to highlight changes in genetic functions included in the microbial metagenome in coral holobionts acclimatized to different OA levels under natural conditions, i.e. in corals collected at different distances from the crater.

## Materials and methods

### Samples collection

Coral specimens were haphazardly collected by SCUBA divers using a hammer and chisel and placed in plastic containers. Corals at the three sampling sites appeared healthy and showed no signs of disease or stress. Mucus was collected using cotton swabs on individual polyps immediately upon coral sampling, once the specimens were brought on the surface [30, 41, 42]. At each site, close to corals collection point, sediments were sampled in 50 mL falcon tubes using a small shovel, and seawater was collected with sterile plastic bottles (2 L per site). All samples (Table 1) were transported in ice to the laboratory and frozen at −80 °C until further processing.

**Table 1:** Summary of *B. europaea* and environmental samples, and features of the sampling sites.

	Environmental parameters		*B. europaea* samples				Environmental samples		
	Sampling site	mean pH	Individuals	Mucus	Soft tissue	Skeleton	Seawater	Sediments	
	Site 1 (control)	8.07	3	3	3	3	1	1	
	Site 2 (mild acidification)	7.87	3	3	3	3	1	1	
	Site 3 (high acidification)	7.74	3	3	3	3	1	1	
Total				9	9	9	3	3	33

### Coral specimens processing and DNA extraction

Coral samples were processed to separate the different coral compartments (mucus, tissue, and skeleton) using standard protocols [30, 43, 44]. Briefly, for mucus samples, the cotton tip of each swab was transferred into a 2 mL Eppendorf tube to which 500 μL of sterile artificial seawater (NaCl 450 mM, KCl 10 mM, CaCl_2_ 9 mM, MgCl_2_·6H_2_O 30 mM, MgSO_4_·7H_2_O 16 mM, pH 7.8) were added. Samples were vortexed for 1 min and sonicated for 2 min, repeating these steps twice and finally vortexing for 1 min. Cotton swabs were then transferred into a new 2 mL Eppendorf tube and the process was repeated. Finally, cotton swabs were discarded, and the suspensions of the same samples were joined and centrifuged at 9000 × *g* for 5 min at 4 °C. Pellets were then stored at −80 °C until further processing. We processed each coral specimen by mechanical fragmentation using an agate mortar to separate coral soft tissue and carbonate skeletal matrix. Coral specimens were transferred into the mortar using sterile forceps and fragmented with the pestle with 10 mL of sterile artificial seawater [30]. Fragmented samples were transferred into a 250 mL beaker and an additional 20 mL of artificial seawater was used to wash the mortar and pestle from residues. The homogenates were incubated at RT for 15 min to allow skeletal fragments to settle. Afterwards, the suspension containing coral tissue was collected and transferred into two 15 mL tubes. Both tubes were centrifuged for 15 min at 9300 × *g* at 4 °C to pellet the coral tissue fraction. The pellets were stored at −80 °C until further processing. For the skeletal fraction, skeleton samples were washed three times with 10 mL of artificial seawater with an incubation of 7 min at RT after each washing to allow the fragments to settle. After discarding the supernatant, skeletal fragments were collected and transferred into a 2 mL Eppendorf tube and kept frozen at −80 °C until further processing.

Total microbial DNA was extracted from each sample using the DNeasy PowerBiofilm Kit (Qiagen, Hilden, Germany) [45]. About 0.1–0.2 g of skeleton samples were weighted and transferred into a PowerBiofilm Bead Tube before being resuspended in 350 mL of Qiagen MBL buffer (guanidine salts). Mucus and tissue pellets were instead resuspended in 350 mL of MBL solution first and then transferred into a PowerBiofilm Bead Tube. DNA extraction was performed with minor adjustments to the manufacturer’s protocol: the homogenization step was performed with a FastPrep instrument (MP Biomedicals, Irvine, CA, USA) at 5.5 movements per sec for 1 min, with a 5 min incubation in ice between treatments, and the elution step was preceded by a 5-min incubation at 4 °C [46]. Extracted DNA samples were quantified with NanoDrop ND-1000 (NanoDrop Technologies, Wilmington, DE, USA) and stored at −20 °C until further processing.

### Environmental samples processing and DNA extraction

Each 2 L of seawater sample was processed via vacuum filtration under sterile conditions using MF-Millipore (Darmstadt, Germany) membrane filters with 0.45-μm pore size (as reported in ref. [47–49]), 47-mm diameter and mixed cellulose esters membrane. Each membrane filter was folded using sterilized forceps and placed into a PowerWater DNA Bead Tube and then stored at −80 °C until further processing. Seawater microbial DNA extraction was carried out using the DNeasy PowerWater Kit (Qiagen, Hilden, Germany) following the manufacturer’s instructions.

Finally, 0.25–0.35 g of sediment samples was weighted into PowerBead Tubes and total microbial DNA was extracted using the DNeasy PowerSoil Kit (Qiagen, Hilden, Germany) following the manufacturer’s instructions with the same minor adjustments described above.

Environmental DNA samples were quantified using NanoDrop ND-1000 (NanoDrop Technologies, Wilmington, DE, United States) and stored at −20 °C until further processing.

### 16S rRNA gene amplification and sequencing

The V3-V4 hypervariable region of the 16S rRNA gene was PCR amplified in 50 uL final volume containing 25 ng of microbial DNA, 2X KAPA HiFi HotStart ReadyMix (Roche, Basel, Switzerland), and 200 nmol/L of microbial 341 F and 785 R primers carrying Illumina overhang adapter sequences [50]. The thermal cycle consisted of 3 min at 95 °C, 25 cycles of 30 s at 95 °C, 30 s at 55 °C, and 30 s at 72 °C, and a final 5-min step at 72 °C [30]. PCR products were purified using Agencourt AMPure XP magnetic beads (Beckman Coulter, Brea, CA, United States). Indexed libraries were prepared by limited-cycle PCR with Nextera technology and cleaned-up as described above. Libraries were then quantified using Qubit 3.0 fluorimeter (Invitrogen), normalized to 4 nM and pooled. Finally, the library pool was denatured with 0.2 N NaOH and diluted to 6 pM with a 20% PhiX control. Sequencing was performed on Illumina MiSeq platform using a 2 × 250 bp paired-end protocol, according to the manufacturer’s instructions (Illumina, San Diego, CA, United States).

### Microbial DNA enrichment, library preparation and shotgun sequencing

Coral DNA samples were subjected to a further processing for shotgun sequencing. Microbial DNA extracted from skeleton and tissue was enriched by selective removal of methylated eukaryotic DNA using the NEBNext Microbiome DNA Enrichment Kit to maximize the protocol efficiency [51] following manufacturer’s instructions. Both enriched DNA and total microbial DNA from mucus samples was quantified using Qubit fluorometer (Invitrogen, Waltham, MA, USA) and DNA libraries were prepared using the QIAseq FX DNA library kit (Qiagen, Hilden, Germany) in accordance with the manufacturer’s instructions. Briefly, 100 ng of each DNA sample were fragmented to a 450-bp size, end-repaired, and A-tailed using FX enzyme mix with the following thermal cycle: 4 °C for 1 min, 32 °C for 8 min, and 65 °C for 30 min. Adapter ligation was performed by incubating DNA samples at 20 °C for 15 min in the presence of DNA ligase and Illumina adapter barcodes. Following, a first purification step with Agencourt AMPure XP magnetic beads (Beckman Coulter, Brea, CA, USA), library amplification with a 10-cycle PCR amplification and a further step of purification were performed. Samples were pooled at equimolar concentrations of 4 nM to obtain the final library. Sequencing was performed on an Illumina NextSeq platform using a 2 × 150-bp paired-end protocol, following the manufacturer’s instructions (Illumina, San Diego, CA, USA).

### Bioinformatics and biostatistics

For the 16S rRNA gene analysis, a pipeline combining PANDAseq [52] and QIIME 2 [53] was used to process raw sequences for a total of 33 samples. The “fastq filter” function of the Usearch11 algorithm [54] was applied to retain high-quality reads (min/max length = 350/550 bp), then binned into amplicon sequence variants (ASVs) using DADA2 [55]. Taxonomy assignment was performed using the VSEARCH algorithm [56] and the SILVA database (December 2017 release) [57]. All the sequences assigned to eukaryotes or unassigned were discarded. Overall, an average sequencing depth of 9046 ± 3764 high-quality reads per sample was obtained for 16S rRNA gene sequencing.

For the shotgun sequencing analysis, raw reads for a total of 27 samples were filtered for PCR duplicates with Picard tool (EstimatedlibraryComplexity) [58]. Next, Trimmomatic (v. 0.39) [59] was adopted to remove adapters and low-quality bases, setting a minimum quality of 20, with reads length ranging from 35 to 151 bp. Fastqc was then applied to examine the quality of the reads prior and after the reads pre-processing steps [60]. Due to the lack of a deposited genome for *B. europaea* in public repositories, and in order to exclude host reads from subsequent analysis, Megahit was used to generate a co-assembly of all the 27 samples, setting a kmer list of 21, 41, 61, 81 and 99 [61]. EukRep (v. 0.6.6) [62] was adopted to classify the contigs in prokaryotic and eukaryotic bins. The reads mapping to the eukaryotic bin (Bowtie2, v. 2.3.5) [63] were identified with Samtools (v 1.9) [64] and eliminated from each sample (BBMap v. 38.22) (http://sourceforge.net/projects/bbmap/). A total of 73,293,344 high-quality microbial reads were retained.

The taxonomic classification, at family, genus and species level was obtained with Kaiju (v. 1.7.0) [65], with greedy mode, match length and match score of 11 and 65 respectively and considering both the paired and unpaired reads for each sample. Next, we performed reads functional annotation per each sample via MetaCV (v. 2059) [66], obtaining KO numbers and their associated KEGG pathway at different functional levels. For each KO, the associated reads were retrieved, and their corresponding taxonomic annotation was collected as obtained from Kaiju. Functional analysis at KO level was also performed by identifying increasing or decreasing trends for genes in samples at different conditions, considering the prevalence of genes among the three replicates from the same site.

Processed reads for 16S rRNA gene sequencing and for metagenomic sequencing are openly available in European Nucleotide Archive (ENA), reference number PRJEB48073.

All statistical analyses were performed using the R software (R Core Team; www.r-project.org - last access: March 2021), v. 3.6.1, with the packages “Made4” [67] and “vegan” (https://cran.r-project.org/web/packages/vegan/index.html). Beta diversity was estimated by computing the Bray-Curtis distance and the data separation in the Principal Coordinates Analysis (PCoA) was tested using a permutation test with pseudo-F ratios (function “adonis” in the vegan package). Wilcoxon rank-sum test was used to assess differences between replicates taken at different sites. *P* values were corrected for multiple testing using the Benjamini–Hochberg method, with a false discovery rate (FDR) ≤ 0.05 considered statistically significant. A procrustean randomized test (function “protest” in the vegan package) was performed to highlight a significant association between the taxonomic and functional profiles on the microbiome across the entire dataset.

## Results

Nine specimens of the coral *Balanophyllia europaea*, 3 sediment samples and 3 seawater samples were collected in July 2019 from three sites (3 coral individuals per site and 2 environmental samples per site, respectively) along a pH gradient at a CO_2_ seep near Panarea Island (Italy). Site 1 (control) mean total scale pH (pH_TS_) is 8.07, corresponding to surface pH of modern oceans; Site 2 (moderate acidification) mean pH_TS_ is 7.87, aligning with a conservative IPCC CO_2_ emission scenario (SSP2-4.5); Site 3 (high acidification) mean pH_TS_ is 7.74 and it is aligned with a worst-case IPCC scenario (SSP3-7.0). Coral samples were processed to separate the 3 different compartments (mucus, soft tissue, and skeleton), obtaining 27 coral samples: 3 mucus, 3 skeleton and 3 tissue samples from each of the 3 sampling sites (Table 1).

The microbiome compositional structure of the total 33 samples was obtained by NGS sequencing of the V3-V4 hyper variable region of the 16S rRNA gene, resulting in 298,545 high-quality reads with an average of 9046 ± 3764 reads per sample. High-quality reads were subsequently normalized to the lowest number of reads per sample (2350), resulting in 8007 Amplicon Sequence Variants (ASVs). Shotgun sequencing analysis was then applied on the 27 coral samples. A total of 179,644,167 paired-end raw reads were generated, with an average of 6,653,487 ± 1,959,146 paired-reads per sample. For the depletion of the eukaryotic DNA, eukaryotic bins were generated, and the reads mapping to the generated bins were filtered out of our dataset (102,070,850 reads). Microbial reads retained after this eukaryotic DNA depletion procedure were 73,293,344. After quality filtering, reads taxonomic classification resulted in a total of 4,174,277 taxonomically assigned reads (average 154,602 ± 241,549 per sample). The reads functional annotation per each sample yielded a total number of functionally classified reads of 72,222,759 (average 2,674,917 ± 3,028,259 per sample).

### *Balanophyllia europaea* overall microbial compositional structure across compartments and acidification conditions

**Fig. 1:**
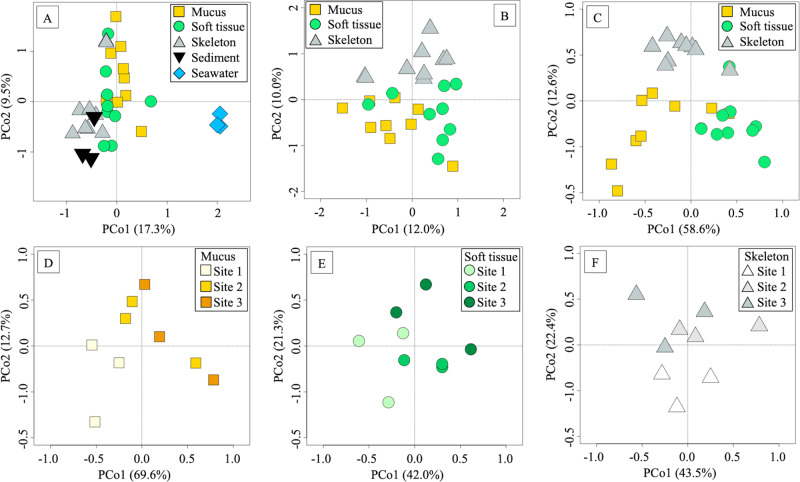
Overall microbiome compositional structure of B. europaea and the surrounding environment. Principal Coordinate Analyses (PCoAs) of the Bray-Curtis distances calculated on microbiome profiles at genus taxonomic level, obtained from 16S rRNA gene sequencing (**A** and **B**) and phylogenetic assignation of metagenomic reads (**C**–**F**), describing different features of the available coral and environmental samples. The first and second principal components (PCo1 and PCo2) are plotted in all graphs and the percentage of variance in the dataset explained by each axis is reported. **A** PCoA based on 16S rRNA gene data showing the structure of B. europaea associated microbiomes compared to the surrounding environment (permutation test with pseudo-F ratio, *p* value = 0.001). **B** PCoA based on 16S rRNA gene data showing B. europaea microbiomes associated to the 3 different coral compartments (permutation test with pseudo-F ratio, *p* value = 0.001). **C** PCoA based on metagenomics data showing B. europaea microbiomes associated to the 3 different coral compartments (permutation test with pseudo-F ratio, *p* value = 0.001). **D**–**F** PCoAs based on metagenomics data comparing B. europaea microbiome in different anatomic compartments in samples collected at different acidified conditions. **A**–**C** legend: mucus samples, yellow squares; soft tissue samples, green circles; skeleton samples, gray triangles; water samples, light blue diamonds; sediment samples, black reversed triangles. PCoA based on metagenomics data comparing B. europaea mucus (**D**), soft tissue (**E**) and skeleton (**F**) microbiomes in corals collected at different acidified conditions. Samples are depicted as symbols (same as in panels **A**–**C**) filled in shades of color from lighter color (Site 1, control site, non-acidified) to darker colors (Site 2, moderate acidification, and Site 3, strong acidification).

*B. europaea* mucus, tissue and skeleton microbiomes constituted separate communities from those of the surrounding environment, in terms of phylogenetic composition at genus level, as shown by the Principal Coordinate Analysis (PCoA) based on the Bray-Curtis distance between 16S rRNA gene profiles (Fig. 1A). Segregation of coral microbiomes was significant with respect to both seawater and sediment microbial communities (permutation test with pseudo-F ratio, *p* value = 0.001), regardless of the coral compartment, although sediment and skeleton samples slightly overlapped on the PCoA plot.

The microbiomes of the 3 coral compartments significantly differed among each other, in terms of phylogenetic composition at the genus level, as observed by computing Bray-Curtis distances among samples using both 16S rRNA gene (Fig. 1B) and shotgun metagenomic taxonomy profiles (Fig. 1C) (permutation test with pseudo-F ratio, *p* value = 0.001 for both). Concordance between metagenomic and 16S rRNA gene data was highlighted by using a procrustean randomized test (“protest”) (*p* value = 0.001, correlation in a symmetric rotation = 0.6028). Taxonomy summary of *B. europaea* microbial family composition based on 16S rRNA gene data is also provided in Supplementary Table S1. The compositional structure of the three compartments, as assigned using Kaiju on metagenomic data at phylum level (Supplementary Fig. S1), was coherent with the available literature on the same coral species [68]. In particular, Proteobacteria was the most abundant phylum in all coral compartments, with a prevalence of Alphaproteobacteria followed by Beta and Gammaproteobacteria, and with Bacteroidetes also listed among the dominant phyla.

Microbiome composition of corals living at different pH conditions along the Panarea gradient (*i.e*., at different distances from the crater) was assessed separately on the 3 anatomic compartments (Fig. 1D–F). Microbial communities associated with mucus from corals taken at the control site (Site 1) and at both the acidified locations (Site 2 and 3) significantly differed in terms of composition, as shown by the separation in the PCoA based on Bray-Curtis distances among samples calculated using phylogenetic data obtained from shotgun metagenomic (Fig. 1D; permutation test with pseudo-F ratio, *p*-value = 0.03). According to our findings, the overall variance of the community structure is attributed at 40% to the anatomic compartment, while site explained the 10% of the total variation.

Significant differences in the microbiome structure were not observed among microbial communities from soft tissue and skeleton (Fig. 1E, F, respectively). Confirming these data, the community composition at the family level in mucus samples showed some noticeable trends of increasing relative abundance in members of the family Burkholderiaceae and decreasing relative abundance in reads assigned to the family Rhodobacteraceae from Site 1 to Site 3 (Supplementary Fig. S2) (Wilcoxon rank sum test of Site 1 compared to Site 2 and 3, *p* value = 0.05 and 0.02 respectively). Conversely, Flavobacteriaceae and Rhodospirillaceae didn’t show any relevant variations among sites.

### Gain and loss of metagenomic functions in *Balanophyllia europaea* living in acidification conditions

**Fig. 2:**
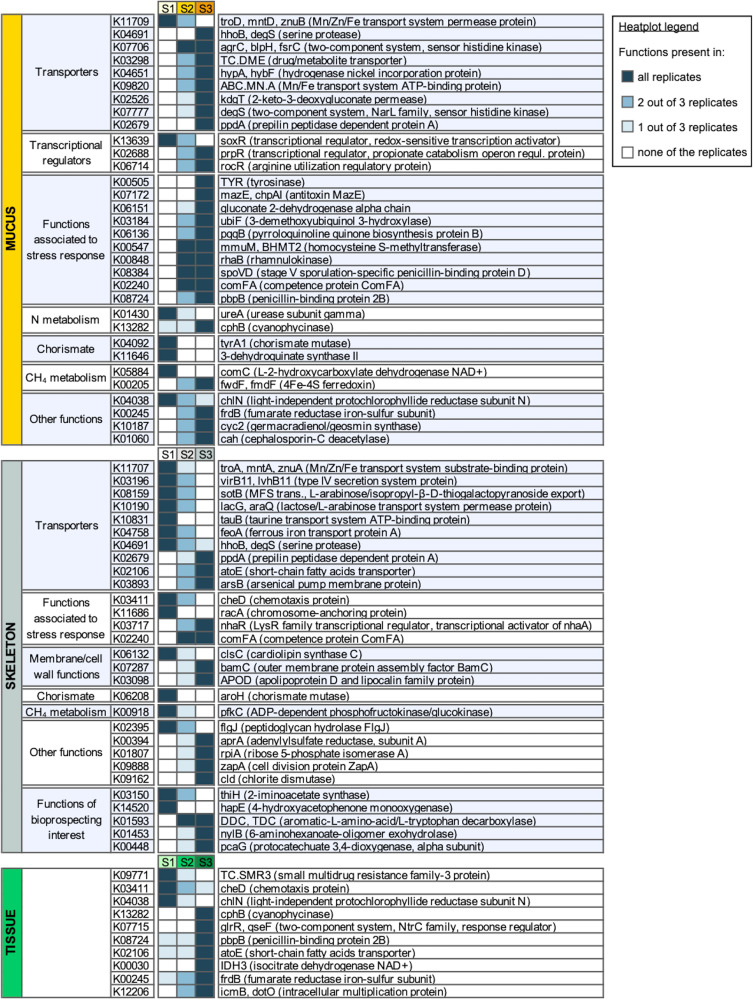
Prevalence-based model for deriving acidification-related gain/loss of metagenomic functions. KEGG orthologs (KO genes) List of KO entries (KEGG orthologs) in the metagenome obtained from the 3 *B. europaea* anatomic compartments (mucus, soft tissue, and skeleton) in different acidification conditions (S1, control site; S2, moderate acidification; S3, high acidification). KO genes showing a prevalence of 100% (i.e., detected in 3 out of 3 replicates) in S1 and 0% (i.e., none of the 3 replicates) in S3 or *vice versa*, are reported, identifying functions lost or gained with acidification, respectively. A heatplot with a color gradient corresponding to the number of replicates in which each KO is detected is provided (see legend top-right for color key). For each KO, listed with KO identification number (left), the KEGG description is given on the right of the heatplot. KO entries are grouped by coherence of involvement in different physiological functions (i.e., genes classified as involved in transcriptional regulation, membrane transport, membrane or cell wall functionality maintenance, stress response, nitrogen (N) metabolism, chorismate pathway, methane (CH_4_) metabolism), as well as by their increasing or decreasing trends among acidification conditions in the same coral compartment, whenever possible.

To highlight changes associated with water acidification on metabolic potential of the coral microbiome, in terms of gain and loss of genetic functions, we focused at the level of KEGG orthologs (KO entries), i.e., groups of genes performing the same function. Starting from a dataset of over 3000 assigned KO, we selected in each tissue the KO entries showing a prevalence of 100% (i.e., detected in 3 out of 3 replicates) in the control site (Site 1) and 0% (i.e., none of the 3 replicates) in the highly acidified site (Site 3) or *vice versa*. The result of the analysis was a prevalence-based model for deriving functions that were under selective pressure by the different pH/pCO_2_ levels at the 3 sites. Indeed, through this reductive approach, we were able to focus on microbial functions that were gained or lost in host grown under low pH/high pCO_2_ levels (Fig. 2).

In mucus and skeleton, a wide set of microbial genes underwent changes in the presence/absence profile of coral specimens collected at highly acidified conditions, whereas in coral soft tissue a smaller number of KO entries showed a clear trend. In particular, mucus and skeleton metagenomes showed changes in prevalence in KOs assigned to proteins involved in transport systems and functions associated to the stress response. For instance, metals transporters (e.g., K11709 in mucus, K11707 and K04758 in skeleton) were lost in samples from the acidified sites, whereas other genes with similar functions appeared (e.g., K09820 in mucus). Other transporters acquired by the microbiome of corals growing in acidified conditions were the transporter ppdA (both in mucus and skeleton) [69], two transporters connected to histidine kinase sensor protein (K07706 and K07777, in mucus) [70], a short chain fatty acid transporter (K02106, in skeleton), and the bacterial outer membrane lipoprotein Blc (K03098, in skeleton), involved in transport of lipids for membrane maintenance [71].

For what concerns stress response, the mucus metagenome of corals growing in highly acidified conditions consistently gained several functionalities, such as the mazE gene (K07172), a tyrosinase function (K00505) involved in production of protective pigments [72], the gluconate 2-dehydrogenase (K06151), the pyrroloquinoline quinone (pqq) biosynthesis protein (K06136), the gene mmuM (K00547), and the rhamnulokinase rhaB (K00848). Moreover, the penicillin-binding protein B2 (K08724), increased its prevalence in tissue samples besides being acquired by mucus samples from the acidified site, and the competence protein ComFA (K02240) is gained by samples from acidified sites in both mucus and skeleton.

Highly acidified conditions were also associated with the gain of the transcriptional regulator nhaR (K03717) in skeleton, whereas, in the same compartment, we observed the loss of the arabinose transport system permease protein (K10190) and of the taurine transport system (K10831).

Most of the above-mentioned functions were detected in 1 or 2 out of 3 replicates in samples taken at Site 2 (Fig. 2), showing how corals growing in moderately acidified waters could represent a transition state towards acclimatization.

Regarding nitrogen (N) metabolism, the urease ureA (K01430) was progressively lost in mucus metagenomes with the increase of acidification, whereas the cyanophycinase cphB (K13282) was gained both in mucus and tissue samples from acidified sites. In order to thoroughly explore N organication and storage pathways, we observed the prevalence of the cyanophycin synthetase (CphA) gene (K03802) and of the Nif gene cluster (*i.e*., genes responsible for N fixation) in the different acidification sites. These genes were consistently detected in all acidification conditions (Supplementary Tables S2 and S3). Hence, the gain of the cphB function points at an acidification-induced gain of potential for N mobilization. Supporting this data, a regulatory protein of arginine utilization (K06714) also appeared in mucus samples from the acidified site.

Other peculiar functions that were lost in acidified conditions (both Site 2 and Site3) were two chorismate mutases (tyrA1, K04092, and aroH, K06208, in mucus and skeleton respectively) and the 3-dehydroquinate synthase (K11646, mucus) (Fig. 2), which are part of the chorismate pathway, involved in the production of aromatic precursors of a wide range of secondary metabolites [73].

We also noticed changes in the prevalence of a few genes involved in functions that could be considered of bioprospecting interest for bioremediation applications, i.e. the genes nylB (K01453) and pcaG (K00448), respectively involved in the nylon degradation pathway [74] and in PAH degradation [75], which were acquired in skeleton samples from the acidified site.

### Functional metagenomic shifts in *Balanophyllia europaea* living in acidification conditions

**Fig. 3:**
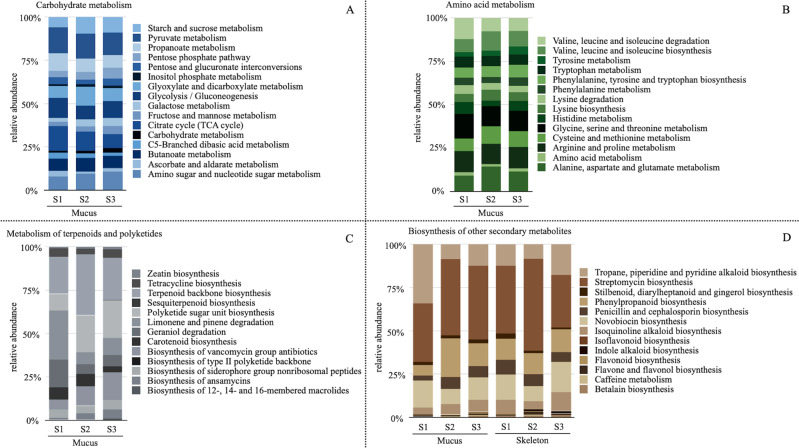
Distribution of functional pathways in selected KEGG networks in *B. europaea* metagenomes. Bar plots representing average relative abundance of KEGG pathways expressed as percentages of reads assigned to specific pathways with respect to the total number of reads assigned to the networks “Carbohydrate metabolism” (KEGG classification 1.1) (**A**), “Amino acids metabolism” (KEGG classification 1.5) (**B**), “Metabolism of terpenoids and polyketides” (KEGG classification 1.9) (**C**), and “Biosynthesis of other secondary metabolites” (KEGG classification 1.10) (**D**). All bar plots refer to the metagenome obtained by mucus samples of *B. europaea* collected at Site 1 (S1), Site 2 (S2) and Site 3 (S3), with the exception of the network “Biosynthesis of other secondary metabolites” (**D**) for which data referring to skeleton samples are also plotted. Color legend is provided for each plot.

Given the above-described gain/loss of metagenomic functions, in particular in the mucus and skeleton metagenomes, we explored shifts in relative abundance of the different pathways within selected KEGG networks possibly connected with the functional variations observed at orthologs level in corals growing at different acidified conditions (Fig. 3). In the mucus metagenome, the network “Carbohydrate metabolism” (KEGG classification 1.1) showed changes in the carbon utilization, with the percentage of reads assigned to the pathways “amino sugar and nucleotide sugar metabolism” and “starch and sucrose metabolism” significantly increasing between the control site and the two acidified sites (Wilcoxon rank sum test of Site 1 vs. Site 2 and 3 together, *p* value = 0.02 and 0.05, respectively). Specifically, the former increased from an average (avg.) of 7.9% in the control to 9.5% and 10.8% in the two acidified sites (Site 2 and Site 3, respectively), whereas the latter increased from avg. 5.9% to 9.8% and 9.0%, respectively. At the same time, we observed a significant decrease in the relative abundance of “glycolysis/gluconeogenesis” pathway, from avg. 11.6% to 8.0% and 9.8% (Wilcoxon test Site 1 vs. Site 2 and 3, *p* value = 0.05). Among the pathways included in the network “Amino acid metabolism” (KEGG classification 1.5), we observed in the mucus metagenome a significant increase in the reads assigned to “cysteine and methionine metabolism” (from avg. 7.4% in the control site to 10.2% and 9.1% in the acidified sites, Wilcoxon test Site 1 *vs*. Site 2 and 3, *p* value = 0.05) and also in the “alanine, aspartate and glutamate metabolism” (from avg. 9.0% in the control site to 14.3% and 11.6% in the acidified sites, Wilcoxon test Site 1 vs. Site 2 and 3, *p* value = 0.05). Finally, we observed changes in the relative abundance of pathways connected to the production or degradation of a wide variety of secondary metabolites (i.e., within the networks “Metabolism of terpenoids and polyketides”—KEGG classification 1.9—and “Biosynthesis of other secondary metabolites”—KEGG classification 1.10). In mucus metagenome, we found the “geraniol degradation” and the “limonene and pinene degradation” pathways to significantly decrease in terms of relative abundance with acidification (from avg. 16.1% to 5.6% and 6.4% and from 28.3% to 6.8% and 9.9% respectively, Wilcoxon test Site 1 vs. Site 2 and 3, *p* value = 0.02 for both), whereas the “polyketide sugar unit biosynthesis” increased with acidification (from avg. 9.6% to 21.3% and 21.6%, Wilcoxon test Site 1 vs. Site 2 and 3 *p* value = 0.02). Furthermore, the percentage of reads mapped in “tropane, piperidine and pyridine alkaloid biosynthesis” pathway decreased with acidification (from avg. 34.1% to 8.7% and 12.4%, Wilcoxon test Site 1 vs. Site 2 and 3 *p* value = 0.05). Moreover, in the skeleton we observed a shift in pathways connected with the synthesis of secondary metabolites, with “stilbenoid, diarylheptanoid and gingerol biosynthesis” pathway decreasing with acidification (from avg. 2.9% to 1.3% and 1.1%, Wilcoxon test Site 1 vs. Site 2 and 3 *p* value = 0.03) and with indole alkaloid biosynthesis increasing with acidification (from avg. 0.04% to 1.0% and 1.4%, Wilcoxon test Site 1 vs. Site 2 and 3 *p* value = 0.02).

## Discussion

Ocean acidification (OA) poses a massive threat to marine ecosystems due to its possible impact on calcifying organisms (reviewed by ref. [76]). In spite of the intensive efforts made lately devoted to exploring the effects of global changes on corals, concerning especially tropical species, our understanding on how their biological and physiological processes may change under OA is still limited (as reviewed by ref. [77, 78]). To this concern, temperate species such as *B. eurapaea* targeted in our study, may represent a valuable model given its more pronounced acclimatization capability with respect to tropical species, being naturally able to accommodate wider seasonal environmental variations (as suggested by ref. [37]). In the present study, we highlighted genetic functions, included in the microbial metagenome of the anatomic compartments of *B. europaea*, changing along with OA, and ultimately identify possible bacteria-related acclimatization processes.

In terms of phylogenetic composition, we observed variations at the family-level in the microbial community associated to the surface mucus of corals with increasing acidification, whereas significant shifts were not observed in soft tissue and skeleton samples, which are confirmed as ecologically distinct habitats, as previously highlighted [79]. This is in line with previous findings on microbiome variations induced by acidification in another temperate but non-zooxanthellate coral species (*i.e*., *Astroides calycularis* [30]). Our observation is also coherent with the fact that mucus niche is a “first line” defense layer, located at the interface between the coral itself and the surrounding environment, as suggested by Shnit-Orland and Kushmaro [80], and more recently confirmed by Pollock et al. [81], whose research on Australian corals pointed at the mucus microbiome as more environmentally responsive than the communities associated to tissue and skeleton.

The phylogenetic shift associated to increasing acidification in the mucus microbial community is accompanied by changes at a functional level. For instance, coherently with recent studies showing modifications of the ion transport system in both tropical and temperate corals subjected to pH variations [82, 83], transporters of small molecules (i.e., metals, short chain fatty acids, and lipids) underwent changes in prevalence in mucus – and in this case also skeleton - samples from corals living in acidified conditions. The mucus microbiome was the one in which a wider and more consistent gain of functions associated to stress response was observed. Among the stress-related functions gained by the mucus of corals living under highly acidified conditions we could find the mazE gene, a toxin-antitoxin system activated during adverse environmental conditions [84], a tyrosinase function involved in production of protective pigments during environmental stress [72, 85], the pyrroloquinoline quinone (pqq) biosynthesis protein that synthesizes a redox cofactor for bacterial dehydrogenases under environmental stress conditions [86], a homocysteine S-methyltransferase (mmuM) previously found upregulated under osmotic stress in corals [87], the gene rhaB that is involved in the response to cell wall and membrane stress in bacteria [88], and the competence protein ComFA that has a role in DNA uptake during horizontal gene transfer [89], which represents an adaptive stress-response mechanism in different bacteria [90]. Moreover, some of the functions acquired with acidification are known to be involved specifically in the resistance to acidic stress in model bacteria; these functions include gluconate 2-dehydrogenase alpha chain [91] and 2-octaprenyl-3-methyl-6-methoxy-1,4-benzoquinol hydroxylase [92]. Finally, additional stress-response functions were gained also by the skeleton microbial community in highly acidified conditions, such as the transcriptional regulator nhaR (K03717), responsible for the osmotic induction of a promoter of the stress-inducible gene osmC [93]. Conversely, in the same compartment, we observed the loss of transporters for important coral osmolytes (arabinose and taurine) used by the metaorganisms to cope with environmental fluctuations [79]. Taken together, these observations point at a possible mechanism of selection of stress-adaptable microbiome components, which might contribute to the coral acclimatization process.

**Fig. 4:**
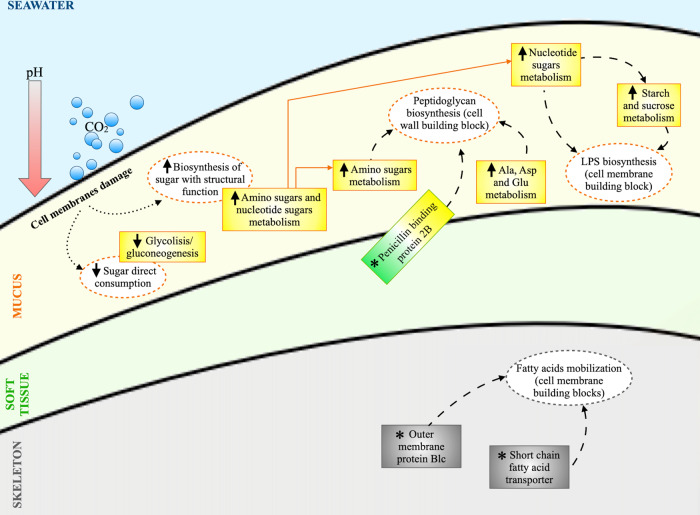
Proposed model of carbohydrate metabolism shift in coral microbiomes under acidification conditions, from direct energy production to structural maintenance pathways. KEGG pathways and KO entries showing acidification related modifications in relative abundance or prevalence, respectively, are reported in rectangles using the same color code of the coral compartment in which variations were observed (mucus, yellow; soft tissue, green; skeleton, gray). Upward and downward arrows indicate that the KEGG pathway increased or decreased in terms of relative abundance with the increasing acidification, respectively. Reported KO entries (*) were detected in all replicates from the highly acidified site while absent in samples from control sites. Cell functions hypothesized to be connected with the variations of KEGG pathways abundance and KO entries detection are reported in dashed circles. Dotted arrows represent the influence of acidification on carbohydrates utilization. Dashed arrows represent connections between observed increasing/decreasing pathways and cell functions. Abbreviations: LPS lipopolysaccharides; Ala Alanine; Asp Aspartate; Glu Glutamate.

Secondly, the microbiome of corals living in acidified conditions showed quantitative shifts in the pathways of carbohydrate metabolism and changes in processes involving metabolites necessary for maintaining protective cell structures, such as lipid membranes and cell walls. Our findings indicate that carbohydrate metabolism in coral microbiome under OA, in particular in the mucus compartment, was subjected to a shift from energy production to maintenance of cell membrane and wall integrity, with a decrease in direct sugar consumption and an increase in structural sugar biosynthesis pathways (Fig. 4). According to our data, the utilization of carbon sources in the mucus underwent a shift in favor of amino and nucleotide sugars metabolism, which are important precursors of the lipidic membranes and cell wall [94, 95], to the detriment of energetic pathways, such as glycolysis/gluconeogenesis. Environmental changes, including lowering pH, might lead to cell membrane damages [96] or alteration in cell structural lipids [97], meaning that membrane bioenergetics and lipid physiology are closely related to the stress response [98]. Since a wide range of nucleotide sugars are required for lipopolysaccharide (LPS) biosynthesis [95], we can assume that changes in environmental conditions might influence the metabolism of nucleotide sugar production by provoking alterations in bacterial cells membranes. Moreover, nucleotide sugars are also essential for sucrose synthesis, with sucrose synthase enzyme having a dual role in producing both UDP-glucose, necessary for cell wall and glycoprotein biosynthesis, and ADP-glucose, necessary for starch biosynthesis [99]. Hence, the increasing trend of both nucleotide sugar pathway and sucrose metabolism that we observed under high acidification is coherent with the strong interconnection between these two pathways and the damages possibly induced by OA. These findings are also supported by the gain of a short chain fatty acid transporter and of an outer membrane protein involved in membrane repair (Blc) in the metagenome of skeleton samples collected under highly acidified conditions. Among their multiple roles, fatty acids are indeed important structural constituents of phospholipids, which are the building blocks of cell membranes [100]. In addition, the ability of Blc to bind several fatty acids and lysophospholipids (LPLs), key inner membrane intermediates of phospholipid metabolism, makes this membrane protein likely involved in cell envelope LPL transport in case of membrane damage [71, 101].

Amino sugars also have an important structural role as components of the prokaryotic cell walls, where they occur in peptidoglycan, LPS, and pseudopeptidoglycan [94]. For example, it has been shown in a model cyanobacterium that peptidoglycan incorporates L-alanine, D-alanine, D-glutamate and meso-diaminopimelate into peptide bridges, which are linked to polymers consisting of alternating amino sugar (acetyl-glucosamine and acetyl-muramate) monomers [95]. This is in line with the increasing relative abundance in the pathway of alanine, aspartate and glutamate metabolism within the amino acids metabolism that we observed with augmented OA, although we could not find a direct link between ocean acidification and cell wall modifications in the available literature. Supporting this possible change in peptidoglycan structure due to highly acidified conditions, we also observed the penicillin-binding protein B2 function, involved in the polymerization of peptidoglycan [102], appearing in mucus and increasing in prevalence in tissue metagenomes under high acidification conditions (Fig. 4).

Finally, in our model of coral acclimatized to low pH, we observed an acidification-induced selection of functions related to Nitrogen (N) mobilization in the mucus metagenome. As OA alters microorganism biogeochemical environment, it is of particular relevance to understand whether and how it is able to affect N cycling in ecologically relevant benthic holobionts [103]. Our results suggest that organic N mobilization is promoted by acidification, especially in the mucus, through the gain of the cyanophycinase function. Cyanophycin is a water-insoluble storage biopolymer acting as N reservoir and synthesized by the enzyme cyanophycin synthetase [104]. Cyanophycinase, responsible for the release of the dipeptide β-aspartyl-arginine from cyanophycin and subsequent hydrolyzation to aspartate and arginine by an isoaspartyl dipeptidase [104–106], only appears in the microbiome associated to corals growing under highly acidified conditions. Accordingly, the appearance of a regulatory protein of arginine utilization in coral mucus growing in acidified sites supports our hypothesis, since arginine is a building block for cyanophycin [107]. On the contrary, N fixation did not show acidification-related modifications in the involved genetic functions, in our Mediterranean coral model, confirming the importance of this pathway for coral survival pointed out by previous studies using ^15^N_2_ tracer technique [108]. Growth and density of Symbiodiniaceae algal symbiont within the coral host is highly dependent on N availability, and N fixation performed by bacteria could contribute to the stability of the coral–algae symbiosis, in particular under sub-optimal scenarios [109]. It is tempting to hypothesize that, with the increased acidification, the N demand in either all or one among the components of the *B. eurapaea* holobiont (*i.e*., the prokaryotic community, the coral host, the symbiotic algae) might increase, and that the gain in terms of functions for N mobilization from storage polymers in the microbial community might be a coping strategy for the sub-optimal environmental conditions.

Studies linking coral N metabolism and environmental variations, in particular heat and eutrophic stresses, have provided a wide array of different, sometimes contrasting, results, depending on the species. For instance, increased ammonia availability allows the maintenance of photosynthesis and calcification rates in the coral *Turbinaria reniformis* under thermal stress [110], whereas an excess of N of anthropogenic origin exacerbates the bleaching reaction to thermal stress in *Acropora* and *Pocillopora* [111]. Pogoreutz et al. [112] proposed a coral bleaching model in which an increased N fixation is synergic with ocean warming in determining the loss of control over the symbiosis with Symbiodiniaceae in *Pocillopora* model. However, the balance in nutrients exchange among bacteria, the coral host, and the zooxanthellae is deemed extremely complex, and it involves mechanisms of N limitation and phosphorous starvation to allow the host to exert control on the photosynthesis in the algal symbiont and maintain the symbiotic homeostasis under changing environmental conditions [112]. To the best of our knowledge, mechanistic studies linking N cycle and tolerance to OA in temperate corals are still unavailable, and our results highlight that any attempt at deepening our knowledge in this field needs to consider the N storage and mobilization pathways and, most importantly, take into account the crosstalk among all the components of the holobiont (coral, algae, and bacteria).

Other peculiar changes involved in N metabolism observed in the mucus metagenome was loss of the urease gene (ureA). Urea has been proposed to represent an important metabolite for coral calcification through degradation by urease [113], which catalyzes the hydrolysis of urea to inorganic carbon and ammonia that are involved in the calcification process [114]. The net calcification rate of *B. europaea* is actually reduced with increasing acidification [25], thus it is possible that a loss in the urease activity might be involved in the process.

In conclusion, the metagenomic changes observed in corals acclimatized to low pH suggest a functional shift able to mitigate the sub-optimal environmental conditions at three different levels. First, at mucus level, the low pH of surrounding water could exert a selective pressure on microbiome composition promoting the acquisition of bacteria genetically equipped for dealing with environmental stress, as demonstrated by the gain of functions related to stress resistance. Secondly, the carbohydrate metabolism of the coral microbiome, especially in the mucus compartment, is affected by acidification in ways that hint at a more efficient maintenance of cell protective structures, confirming the importance of membrane bioenergetics in connection to the response to acidification, as previously reported in different contexts [98]. Thirdly, acidification promotes the selection of genetic functions that can respond to variations in nitrogen needs at the holobiont level possibly in relation to its control over the algal symbiont. Our results point at the importance to consider the crosstalk among all the three components (coral host, symbiotic algae, and bacterial communities) of the holobiont to further unveil nitrogen-involving processes that allow photosynthetic corals to maintain their functionality under adverse environmental conditions.

Our study expands the current knowledge on processes of coral acclimatization to OA and confirms that temperate corals represent a promising model of microbiome adaptation. When confirmed by future mechanistic studies, also microbiome transplants in controlled environment, the processes hypothesized in the present work represent an important step towards a holistic comprehension of the tripartite crosstalk between coral host, symbiotic algae and bacterial communities, as well as a deepened understanding on how this relationship changes under environmental variations allowing for the survival and health of these ecosystem forming holobionts in the forthcoming oceans.

## Supplementary Information

Supplementary Information

## Data Availability

The data that support the findings of this study (processed reads for 16S rRNA gene sequencing and for metagenomic sequencing) are openly available in European Nucleotide Archive (ENA), reference number PRJEB48073.
