# Correction to: Metagenomic shifts in mucus, tissue and skeleton of the coral *Balanophyllia europaea* living along a natural CO_2_ gradient

**DOI:** 10.1038/s43705-022-00165-w

**Published:** 2022-08-30

**Authors:** Giorgia Palladino, Erik Caroselli, Teresa Tavella, Federica D’Amico, Fiorella Prada, Arianna Mancuso, Silvia Franzellitti, Simone Rampelli, Marco Candela, Stefano Goffredo, Elena Biagi

**Affiliations:** 1 Unit of Microbiome Science and Biotechnology, Department of Pharmacy and Biotechnology, University of Bologna, via Belmeloro 6, 40126 Bologna, Italy; 2 Fano Marine Center, The Inter-Institute Center for Research on Marine Biodiversity, Resources and Biotechnologies, viale Adriatico 1/N, 61032 Fano, Pesaro Urbino Italy; 3 Marine Science Group, Department of Biological, Geological and Environmental Sciences, University of Bologna, via Selmi 3, 40126 Bologna, Italy; 4 Animal and Environmental Physiology Laboratory, Department of Biological, Geological and Environmental Sciences, University of Bologna, via Sant’Alberto 163, 48123 Ravenna, Italy

**Keywords:** Metagenomics, Microbial ecology, Climate-change ecology

Correction to: *ISME Communications* 10.1038/s43705-022-00152-1, published online 05 August 2022

In the original version of this article, the given and family names of Giorgia Palladino, Erik Caroselli, Teresa Tavella, Federica D’Amico, Fiorella Prada, Arianna Mancuso, Silvia Franzellitti, Simone Rampelli, Marco Candela, Stefano Goffredo, and Elena Biagi were incorrectly structured. The name was displayed correctly in all versions at the time of publication

The original article has been corrected.

