# Supplementary material for: Metagenomic shifts in mucus, tissue and skeleton of the coral Balanophyllia europaea living along a natural CO2 gradient
Source: ISME Commun. 2022 Aug 5;2:65. doi: 10.1038/s43705-022-00152-1 (PMC9723718; doi:10.1038/s43705-022-00152-1)
Supplement: Supplementary file 1 — Supplementary Information [file 43705_2022_152_MOESM1_ESM.pdf]

Supplementary Information

Metagenomic shifts in mucus, tissue and skeleton of the coral *Balanophyllia europaea* living along a natural CO_2 gradient

Palladino Giorgia[1,2]*, Caroselli Erik[2,3]*, Tavella Teresa[1], D'Amico Federica[1], Prada Fiorella[2,3], Mancuso Arianna[2,3], Franzellitti Silvia[2,4], Rampelli Simone[1], Candela Marco[1,2]†, Goffredo Stefano[2,3]†, Biagi Elena[1,2]

[1] Unit of Microbiome Science and Biotechnology, Department of Pharmacy and Biotechnology, University of Bologna, via Belmeloro 6, 40126 Bologna, Italy

[2] Fano Marine Center, the Inter-Institute Center for Research on Marine Biodiversity, Resources and Biotechnologies, viale Adriatico 1/N, 61032 Fano, Pesaro Urbino, Italy

[3] Marine Science Group, Department of Biological, Geological and Environmental Sciences, University of Bologna, via Selmi 3, 40126 Bologna, Italy

[4] Animal and Environmental Physiology Laboratory, Department of Biological, Geological and Environmental Sciences, University of Bologna, via Sant'Alberto 163, 48123 Ravenna, Italy

*These authors contributed equally

†Corresponding authors: *marco.candela@unibo.it*; *s.goffredo@unibo.it*

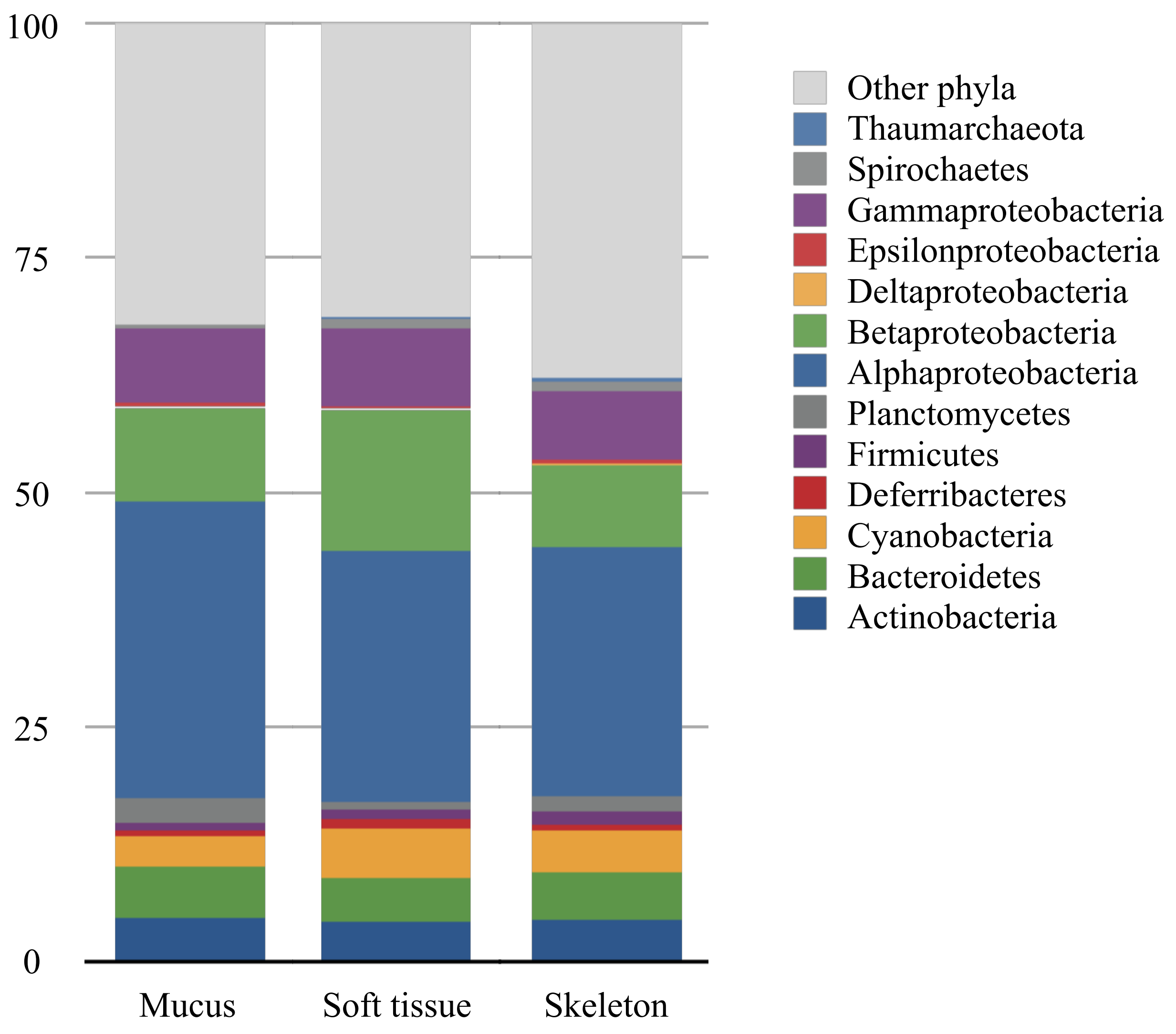

Supplementary Figure S1 – *B. europaea* microbiomes composition at phylum level. Bar plots of the average microbiome composition at the phylum (and class for Proteobacteria) taxonomic level in the 3 different *B. europaea* anatomic compartments (mucus, soft tissue and skeleton). Composition is expressed as relative abundance (%) calculated on the phylogenetically assigned metagenomic reads. The dominant phylum in all tissues is represented by Proteobacteria, with the highest abundance of Alphaproteobacteria (mucus, 31.5%; soft tissue, 26.6%; skeleton, 26.5%), followed by Betaproteobacteria (10%, 15.1% and 8.6%) and Gammaproteobacteria (8.0%, 8.3% and 7.5%). Bacteroidetes is also present as dominant phylum (5.5%, 4.6% and 5.1%).

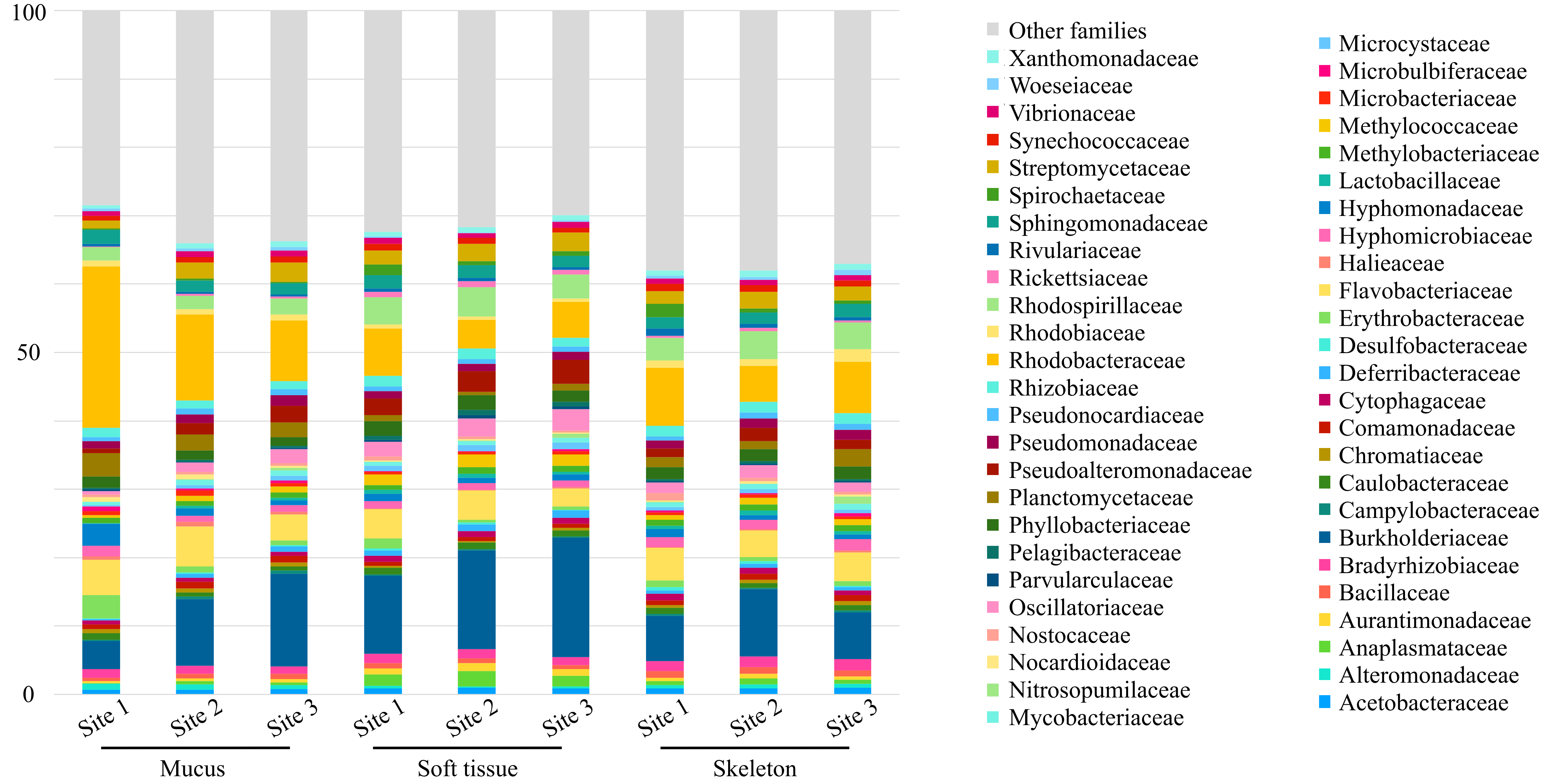

Supplementary Figure S2 – *B. europaea* microbial composition at family level. Bar plots of the average microbiome composition at family taxonomic level in the 3 different *B. europaea* anatomic compartments (mucus, soft tissue and skeleton) and in the 3 different acidification conditions (Site 1, Site 2 and Site 3, corresponding to control conditions, mild acidification and high acidification, respectively). Compositions is expressed as relative abundance (%) calculated on the phylogenetically assigned metagenomic reads. In mucus samples, it can be observed a trend of increasing relative abundance of the family *Burkholderiaceae* from Site 1 to Site 2 and 3 (relative abundance 4.1%, 9.8% and 13.5% respectively) and of decreasing relative abundance of the family *Rhodobacteraceae* (relative abundance 23.6%, 12.6% and 8.1%).

Supplementary Table S1 – Taxonomic summary of *B. europaea* microbial family composition based on 16S rRNA gene data. Table reporting the average relative abundance (r.a.) percentage of microbiome composition at family taxonomic level based on 16S rRNA gene data in the 3 different *B. europaea* anatomic compartments (mucus, soft tissue and skeleton). Avg = average, SD = standard deviation.

Bacterial family (r.a. %)	Mucus		Skeleton		Soft tissue	
	Avg	SD	Avg	SD	Avg	SD
Acidobacteria;Subgroup9;unc	0.39	0.93	0.23	0.60	0.17	0.33
Acidimicrobiaceae	0.66	0.76	0.90	1.47	0.32	0.65
Acidimicrobiales;unc	0.29	0.30	0.97	2.02	0.04	0.11
Alphaproteobacteria_Inc_Sedis	1.56	1.79	0.51	0.39	1.22	0.88
Alphaproteobacteria;Other	0.56	1.02	2.39	2.66	0.62	0.81
Alteromonadales;Other	0.47	1.42	0.00	0.00	0.00	0.00
Bacteroidetes;Other	0.26	0.73	0.29	0.34	0.17	0.30
Bdellovibrionaceae	0.17	0.16	0.28	0.29	0.47	0.77
Burkholderiaceae	0.98	1.87	0.03	0.05	6.26	18.79
Campylobacteraceae	0.08	0.15	0.02	0.06	0.43	1.21
Chlamydiales;Other	0.50	0.44	0.78	0.60	1.08	0.85
Clostridiaceae1	2.74	8.07	0.02	0.07	0.04	0.13
Clostridiales;FamXII	0.67	2.00	0.00	0.00	0.07	0.20
Clostridiales;FamXIII	0.42	1.25	0.00	0.00	0.00	0.00
Clostridiales;Other	0.09	0.15	3.06	5.52	1.25	2.60
Coriobacteriaceae	0.59	1.77	0.01	0.03	0.00	0.00
Coxiellaceae	0.50	0.96	0.09	0.14	0.32	0.50
Cyanobacteria;SubsI;FamI	0.82	1.17	0.10	0.19	0.03	0.09
Cyanobacteria;SubsII;FamII	0.15	0.20	0.60	1.21	0.13	0.29
Cyanobacteria;SubsII;Other	0.00	0.00	0.66	1.36	0.45	1.20
Cyanobacteria;SubsIII;FamI	2.10	5.71	2.54	2.53	0.34	0.58
Deltaproteobacteria;Sva0485	0.00	0.00	0.04	0.13	0.34	0.98
Enterobacteriaceae	0.74	1.81	0.06	0.18	0.79	1.50
Erythrobacteraceae	1.58	2.20	0.61	1.18	0.65	1.10
Flammeovirgaceae	1.36	0.93	7.48	5.23	1.69	1.19
Flavobacteriaceae	4.61	3.62	5.16	4.23	14.62	11.98
Fusobacteriaceae	0.20	0.52	0.15	0.44	1.16	3.48
Gammaproteobacteria;KI89A clade	0.11	0.15	0.12	0.18	0.34	0.72
Gammaproteobacteria;Other	3.57	3.71	3.05	1.48	3.52	2.60
Gracilibacteria;unc	0.47	0.85	0.01	0.04	0.14	0.31
Halieaceae	1.12	1.50	0.44	0.38	0.35	0.39
Helicobacteraceae	6.22	9.96	0.10	0.20	0.56	0.64
Hyphomicrobiaceae	0.50	0.76	2.88	2.64	0.43	0.35
Hyphomonadaceae	1.60	2.32	1.95	2.43	1.00	1.74
Idiomarinaceae	0.81	1.22	0.00	0.01	0.02	0.07
Lachnospiraceae	1.00	2.60	0.50	0.82	1.06	1.83
Moraxellaceae	0.88	1.07	0.00	0.00	0.00	0.00
Myxococcales;P3OB-42	0.09	0.13	1.19	2.55	0.56	0.79
Oceanospirillaceae	0.02	0.05	0.25	0.68	0.02	0.03

Bacterial family (r.a. %)	Mucus		Skeleton		Soft tissue	
	Avg	SD	Avg	SD	Avg	SD
Oceanospirillales;SUP05cluster	0.04	0.11	0.93	2.75	0.06	0.18
Oceanospirillales;Other	0.28	0.77	0.34	0.60	0.78	1.51
Parvularculaceae	0.50	0.39	0.80	0.79	0.82	1.91
Peptostreptococcaceae	2.73	7.63	0.09	0.25	0.63	1.76
Phycisphaerae;CCM11a;unc	0.00	0.00	0.01	0.04	0.28	0.85
Phycisphaeraceae	0.43	0.39	1.48	0.78	0.50	0.49
Phyllobacteriaceae	3.15	6.05	2.67	1.68	2.35	1.59
Planctomycetaceae	7.39	4.03	9.72	3.06	4.52	1.22
Porphyromonadaceae	0.78	2.34	0.00	0.00	0.00	0.00
Proteobacteria;Other	0.07	0.10	0.01	0.04	0.70	1.76
Pseudoalteromonadaceae	0.47	0.85	0.04	0.09	1.01	2.28
Pseudomonadaceae	0.82	1.86	0.21	0.32	0.30	0.85
Rhizobiales;Other	4.03	6.57	3.22	2.19	2.85	3.36
Rhodobacteraceae	11.50	6.04	6.13	6.17	5.74	4.41
Rhodobiaceae	0.27	0.69	0.97	1.58	0.30	0.50
Rhodospirillaceae	1.48	1.73	8.58	8.46	5.18	4.95
Rhodospirillales_Inc_Sedis	0.26	0.36	0.80	1.62	0.27	0.45
Rhodospirillales;Other	1.30	2.61	3.49	6.08	0.87	0.81
Rickettsiales;Other	0.08	0.12	0.01	0.02	0.46	0.79
Rikenellaceae	0.26	0.78	0.00	0.00	0.00	0.00
Ruminococcaceae	0.60	1.15	0.01	0.02	0.40	0.97
Saprospiraceae	0.50	0.47	0.54	0.63	0.78	1.52
SAR324clade(Mar_gr_B);Other	0.06	0.13	0.09	0.12	0.36	1.01
SBR1093;unc	0.33	0.44	0.40	0.62	0.42	0.76
Shewanellaceae	0.04	0.09	0.06	0.13	0.58	1.47
Sphingomonadales;Other	0.92	2.72	0.00	0.00	0.00	0.00
Spirochaetaceae	0.02	0.06	4.94	11.07	5.78	12.24
Spiroplasmataceae	0.00	0.00	0.00	0.00	5.23	14.28
Streptomycetaceae	0.08	0.20	0.01	0.03	0.65	1.07
Synergistaceae	0.25	0.74	0.00	0.00	0.00	0.00
Syntrophaceae	0.00	0.00	0.00	0.01	0.43	0.94
Thiotrichaceae	1.46	2.53	0.36	0.70	0.44	0.74
TM6(Dependentiae);unc	0.02	0.06	0.44	1.13	0.35	0.81
Verrucomicrobiales;DEV007	0.94	0.52	1.00	0.71	0.26	0.33
Verrucomicrobiaceae	2.13	1.80	0.62	0.49	0.33	0.37
Verrucomicrobia;Other	0.09	0.17	0.42	0.50	0.33	0.81
Vibrionaceae	0.49	0.76	0.18	0.30	2.77	5.16
Xanthomonadales;JTB255_mar_benthic_gr	1.42	1.07	2.63	0.99	1.21	1.05
Other_families	15.93	6.67	11.29	4.57	11.39	5.01

Supplementary Table S2 - Variations in the abundance of cyanophycin synthetase (CphA) gene (K03802) in the metagenomes of all coral compartments in individuals collected at different acidification sites. The values of relative abundance (r.a.) are calculated on the total number of reads assigned to KOs (i.e., number of reads assigned to K03802 divided by the sum of all reads in a given sample).

		Compartment	K03802 (r.a. %)			Compartment	K03802 (r.a. %)			Compartment	K03802 (r.a. %)
Site 1 – Control	Individual 1	Mucus	0.0058	Site 2 – Mild acidification	Individual 1	Mucus	0.0192	Site 3 – High acidification	Individual 1	Mucus	0.0092
		Soft tissue	0.0248			Soft tissue	0.0017			Soft tissue	0.0075
		Skeleton	0.0292			Skeleton	0			Skeleton	0.0298
	Individual 2	Mucus	0.0013		Individual 2	Mucus	0.0112		Individual 2	Mucus	0.0091
		Soft tissue	0.0107			Soft tissue	0			Soft tissue	0.0019
		Skeleton	0.0153			Skeleton	0.0261			Skeleton	0.0067
	Individual 3	Mucus	0.0447		Individual 3	Mucus	0.0017		Individual 3	Mucus	0.026
		Soft tissue	0			Soft tissue	0.003			Soft tissue	0.0046
		Skeleton	0.0126			Skeleton	0.0587			Skeleton	0.0124

Supplementary Table S3 - Variations in the abundance of genes associated to the Nif (nitrogen fixation) regulon in metagenomes of all coral compartments in individuals collected at different acidification sites. The values of relative abundance (r.a.) are calculated on the total number of reads assigned to KOs (i.e., number of reads assigned to each Nif-associated KO divided by the sum of all reads in a given sample). The correspondence between KO numbers and genes is the following: K02584 - Nif-specific regulatory protein; K02585 - Nitrogen fixation protein NifB; K02586 - Nitrogenase molybdenum-iron protein alpha chain; K02587 - Nitrogenase molybdenum-cofactor synthesis protein NifE; K02588 - Nitrogenase iron protein NifH; K02591 - Nitrogenase molybdenum-iron protein beta chain; K02592 - Nitrogenase molybdenum-iron protein NifN; K02594 - Homocitrate synthase NifV; K04488 - Nitrogen fixation protein NifU and related proteins.

		Compartment	K02584	K02585	K02586	K02587	K02588	K02591	K02592	K02594	K04488
Site 1 – Control	Individual 1	Mucus	0.0019%	0.0011%	0.0036%	0.0038%	0.0011%	0.0036%	0.0019%	0.0016%	0.0011%
		Soft tissue	0.0215%	0.0148%	0.0101%	0.0228%	0.0020%	0%	0.0215%	0%	0.0040%
		Skeleton	0.0438%	0.0310%	0.0082%	0.0018%	0.0018%	0%	0.0100%	0%	0.0091%
	Individual 2	Mucus	0.0057%	0.6320%	0.0018%	0.0037%	0.0024%	0.0024%	0.0019%	0.0067%	0%
		Soft tissue	0.0107%	0.0164%	0.0100%	0.0064%	0.0014%	0.0136%	0.0157%	0.0050%	0.0050%
		Skeleton	0%	0%	0%	0%	0%	0%	0%	0%	0%
	Individual 3	Mucus	0.0147%	0.0018%	0.0159%	0.0196%	0.0184%	0.0257%	0.0037%	0.0159%	0.0098%
		Soft tissue	0%	0.0148%	0%	0.0485%	0%	0.0138%	0%	0%	0%
		Skeleton	0.0396%	0%	0.0108%	0.0126%	0.0459%	0.0027%	0%	0%	0%
Site 2 – Mild acidification	Individual 1	Mucus	0.0322%	0.0397%	0.0185%	0.0082%	0.0034%	0.0041%	0.0110%	0.0151%	0.0171%
		Soft tissue	0.0017%	0.0103%	0.0017%	0.0361%	0%	0.0456%	0.0103%	0.0112%	0.0103%
		Skeleton	0.0589%	0.0115%	0.0390%	0%	0.0115%	0.0269%	0.0115%	0%	0%
	Individual 2	Mucus	0.0284%	0.0022%	0.0203%	0.0081%	0%	0.0184%	0.0066%	0.0203%	0.0137%
		Soft tissue	0.0469%	0%	0%	0.0517%	0.0065%	0.0065%	0.0129%	0%	0.0016%
		Skeleton	0%	0.0095%	0.0165%	0%	0.0046%	0%	0%	0.0095%	0.0211%
	Individual 3	Mucus	0%	0%	0%	0%	0.0017%	0.0017%	0%	0.0017%	0.0083%
		Soft tissue	0.0161%	0.0030%	0%	0.0030%	0%	0.0030%	0.0030%	0%	0.0131%
		Skeleton	0%	0.0132%	0.0010%	0.0010%	0%	0.0122%	0.0243%	0.0435%	0.0851%
Site 3 – High acidification	Individual 1	Mucus	0.0031%	0.0010%	0.0212%	0.0212%	0.0010%	0.0031%	0.0041%	0.0092%	0.0256%
		Soft tissue	0.0115%	0.0020%	0%	0.0075%	0.0040%	0.0095%	0%	0.0075%	0.0055%
		Skeleton	0.0088%	0.0026%	0%	0%	0%	0.0175%	0.0079%	0.0070%	0.0079%
	Individual 2	Mucus	0.0155%	0.0155%	0.0255%	0.0091%	0.0027%	0.0255%	0.0027%	0.0428%	0%
		Soft tissue	0.0019%	0%	0.0055%	0%	0%	0.0055%	0.0019%	0%	0%
		Skeleton	0.0208%	0.0208%	0%	0%	0%	0.0208%	0%	0%	0%
	Individual 3	Mucus	0.0318%	0.0034%	0.0034%	0%	0.0134%	0.0042%	0.0151%	0.0017%	0.0184%
		Soft tissue	0.0158%	0.0164%	0.0053%	0.0184%	0.0066%	0.0099%	0.0197%	0.0092%	0.0132%
		Skeleton	0.0770%	0.0124%	0%	0%	0%	0.0028%	0.0289%	0.0422%	0.0298%
